# Supplementary material for: Neutrophil Elastase, Neuron-Specific Enolase, and S100B Protein as Potential Markers of Long-Term Complications Caused by COVID-19 in Patients with Type 2 Diabetes Mellitus (T2DM) and Advanced Stage of Diabetic Nephropathy (NfT2DM)—Observational Studies
Source: Int J Mol Sci. 2024 Nov 2;25(21):11791. doi: 10.3390/ijms252111791 (PMC11546864; doi:10.3390/ijms252111791)
Supplement: Supplementary file 1 [file ijms-25-11791-s001.zip › ijms-3229766-supplementary.pdf]

Table S1. Data of patients with T2DM from whom serum and plasma were collected before COVID-19.

No.	AGE [years]	sex	Procalcitonin (0-0.05 ng/mL)	Troponin (0 - 15.6)	PTH (12 - 88 pg/ml)	CRP ultrasensitive (0 - 5 mg/l)	LDL (0-135 mg/dL)	HDL (>40 mg/dL)	Total chol (130-200 mg/dL)	TG (0 - 150 mg/dL)	Glucose (70 - 100 mg/dl)	HbA1c (4-6.0 %)	bilirubine (0.2 - 1.2 mg/dL)	potassium (3.5 - 5.1 mg/dl)	TSH 3rd Generation (0.55 - 4.78)	Total protein (6.6 - 8.3 g/dl)	Urea (11 - 43 mg/dl)	Creatinine (0.57-1.1 mg/dL)	eGFR (>90)	Hypertension	Ischemic heart disease (IHD)	Atrial fibrillation (AF)	Myocardial infarction (MI)	Heart failure	Stroke	Generalized atherosclerosis	Micro-angiopathy	Macro-angiopathy	Nephropathy	Neuropathy	Retinopathy	Hypothyroidism	Secondary hyper-
1	69	M					133	42	196	106	102	6.1	1.2	4.7	1.414	6	44	1.36	55	YES					YES	YES							
2	68	M	0.06	5.3		9.73	93	51	186	209	126	5.5	0.7	4.94	1.556	7.7	49	1.23	62	YES	YES	YES						YES	YES				
3	56	M	0.08	5.3		9.81	62	39	124	115	197	10	0.6	4.35	1.574	7.5	39	1.27	62	YES					YES	YES		YES	YES				
4	78	M	2.8	7.9		235.33	107	44	183	161	230	8.4	0.5	4.2	4.9927	7.2	48	1.13	67	YES	YES		YES			YES		YES	YES			YES	
5	63	M				3.66	187	46	263	151	186	9.5		4.4	4.992	7.2		0.91	89	YES									YES				
6	69	M	0.02			2.03	72	44	129	67	140	7.8	0.6	4.6	1.393	7.1	40	1	79							YES			YES				
7	50	F	0.01	6.8		1.93	116	51	187	102	108	6.2	0.9	4.27	7.407		19	0.86	74										YES				
8	55	M				1.37	151	36	225	190	214	10.2	0.6	4.19	0.992	6.6	21	0.69	127	YES						YES			YES				
9	60	M				1.37	129	48	235	290	118	9.6	0.8	3.97	1.639	6.6			64	YES									YES				
10	64	M	0.26			21.78	47	34	96	74	130	7.5		5.7	3.248	6.4	49	6.44	9	YES	YES					YES	YES	YES	YES		YES		
11	77	M	0.32	100.7		224.11	38	41	102	115	126	7.1	0.4	3.49	0.728	6.8	69	1.85	38	YES	YES					YES			YES				
12	31	M				60.74	142	54	225	146	188	8.2	0.9	3.69	3.087	7.9	10	0.73	133	YES									YES			YES	
13	73	M	0.02	11		10.14	62	17	139	298	117	7.5	0.5	4.1	1.118	6.6	34	1.1	70	YES						YES		YES	YES				
14	62	F	0.05			3.83	62	60	149	134	195	10.6	0.6	4.58	1.797	7.2	38	0.94	64	YES	YES			YES	YES	YES	YES	YES					
15	80	M				1.26	48	38	115	145	126	6.7	0.8	4.88	1.409		34	0.91	85	YES	YES	YES		YES	YES	YES			YES			YES	
16	69	M		10.4		0.96	145	64	246	184	143	7.3		4.18	0.465	7.2		0.96	83	YES	YES					YES		YES	YES				
17	50	F				1.52	62	57	192	365	155	8.8	0.5	3.99	2.009	6.5	30		84	YES									YES	YES		YES	
18	66	F	0.05			47.27	66	37	136	166	166	6.5	0.4	4.28	1.557	6.3	43	1.42	39	YES						YES		YES	YES				
19	75	M			23	2.44	62	39	133	162	225	10	0.7	3.92	0.17	7.2	124		22	YES	YES	YES	YES	YES		YES			YES				YES
20	67	M	0.02	24.5		52.28	63	32	121	126	223	7.8	0.6	5.29	3.45	6.3	68	1.75	41	YES	YES					YES			YES				
21		M																															
22	31	M				5.97	155	42	221	122	122	7.9		4.08	3.277	8.2	15	0.93	101	YES									YES			YES	
23		M	1			5.64	120	31	201	249	173	5.6	0.6	4.31	3.716	6.3	125	1.87	40	YES				YES									
24	62	M	0.07	19.3		20.69	40	37	114	147	174	7.7	0.3	4.46	2.919	6	35	0.99	81	YES	YES		YES				YES		YES				
25	56	M				0.48	131	49	230	248	149	6.8		4.03	0.879	6.4	20	0.68	128	YES						YES	YES	YES	YES				
26		M																											YES				

27	64	M	0.05			2.55	101	67	191	115	172	8.2		4.2	2.278	6.7	20	0.82	100	YES		YES				YES							
28	61	F	0.02	7		2.85					133	8.1	0.6	3.95	2.772	6.7	51	0.78	78	YES		YES				YES	YES	YES					
29	72	M	0.14			27.44	162	48	265	275	160.3	13.4	0.6	4.67	1.739	7.3	25	1.28	59	YES						YES			YES				
30	66	M	0.08	18.6		84.30	69	43	146	168	289	12.9	0.8	5.04	0.666	6.4	64	1.35	56		YES		YES	YES	YES	YES							
31	64	F				1.52	103	58	186	127	155	9.4		3.88	1.715	7.6	37	1.21	47	YES		YES			YES				YES	YES			
32	44	F				3.9	132	58	210	99	155	8.1		3.93	0.437	7.8	27	0.66	103	YES									YES				
33	31	M				9.88					107			3.7	2.22	8.3	19	0.79	122	YES												YES	
34	52	M				1.71		46	337	420	118	8.4	0.6	4.14	3.335	7.5	43	1.14	71	YES						YES	YES	YES	YES		YES		
35	56	M		8		6.92	78	44	167	223	110	8.4	2.2	4.12	1.171	7.8	36	1.08	75	YES					YES	YES		YES			YES		
~~36~~																																	
37	76	F	0.02			42.64	188	62	296	231	303	6.4	0.4	5.03	0.444	9.1	40	1.06	53	YES					YES				YES				
38	58		0.04			4.71		34	180	433	267	12.6	1	4.77		7.7	38	0.89	93	YES	YES		YES	YES		YES	YES	YES					
39	61		0.08	9.1		62.5	70	18	119	157	138	9.6	0.8	4.21	0.396	5.7	57	1.05	77	YES							YES	YES	YES		YES		
40	63					2.05	114	38	180	138	110	6.3		4.4	0.621	6.3	31	0.92	88						YES								
41	71		0.03			5.02	117	37	219	325	141	8.2	1	3.71	0.894	7.1	32	0.95	83	YES						YES	YES	YES					
42	87		0.03			36.83	54	36	114	123	59	6.8		4.01	2.711	5.2	28.5	1.02	73	YES						YES		YES	YES				
43	74					37.5	40	21	98	186	91		0.5	4.33	1.387	6.1	30		55	YES		YES				YES		YES					
44	78		0.19			2.81	48	39	101	70	182	8.2		4.14	1.397	7.6	101	1.74	40	YES	YES	YES		YES		YES	YES	YES	YES		YES		
45	70					12.15	81	30	156	223	266	12.8	0.5	4.8	0.07	6.5	30	1.4	53	YES						YES		YES				YES	
46	65		0.15	1.9		40.5	143	37	208	142	146	7.5	0.8	4.75	0.811	7.1	50	1.41	54	YES									YES				
47	71		0.02			3.84	145	52	219	111	170	8.7	0.5	4.63		7.3	30	1	78	YES						YES							
48	68	F				1.7	111	51	190	142	196	7.8			2.758	7.9	54	1.55	35													YES	
49	67					2.13	111	34	208	316	199	9.5	0.9	4.69	0.117	7	59	1.31	58	YES						YES		YES					YES
50	77	F			125.6	3.16	188	50	286	240	186	8.7	0.4	4.88	4.797	6.9	31	1.12	50	YES	YES				YES	YES		YES	YES				
51	72	F	0.02	5		1.97	53	52	149	221	176	7.5	1.5	4.38	4.891	7.5	61	1.52	50	YES			YES		YES	YES	YES	YES				YES	
52	82	F				3.52	118	52	193	115	143	6.9		4.49	0.742	6.7	55	1.27	43	YES						YES							
53	65	F	0.02	2.2		22.9	128	40	189	107	132	6.2	0.5	3.82	0.219	6.4	24	0.59	108	YES						YES	YES	YES					YES
54	85	F	0.07			28.97					128	12.4		4.11	0.709		22	0.65	92	YES						YES							
55	74	F	0.06	132.7		5.93	158	50	243	175	152	6	0.3	4.2	2.524	6	113	2.27	22	YES				YES		YES			YES				
56	52	F	0.07		98.8	27.95	76	39	172	286	91	5.5		3.18	1.443	6.4	59.5	1.26	47	YES	YES		YES	YES		YES							
57	62		0.02			22.48	85	25	136	128	83	6.6	0.4	4.42	1.477	8.7	29	1.03	78	YES										YES			

58	61		0.01	6	0.83	69	65	140	29	117	9.4	1.1	4.21	1.153	6.5	47	0.99	89	YES	YES		YES	YES	YES	
59	55				6.09	66	46	153	204	87	6.4	0.5	4.22	0.867	6.5		1.11	73							
60	83	F	0.08		49.535	90	29	140	126	236	7		4.77	0.078	6.8	68	1.17	47	YES		YES	YES			YES

Table S2. Data of patients with T2DM from whom serum and plasma were collected after COVID-19.

No.	AGE [years]	sex	Procalcitonin (0-0.05 ng/mL)	Troponin (0 - 15.6)	PTH (12 - 88 pg/ml)	CRP ultrasensitive (0	LDL (0-135 mg/dL)	HDL (>40 mg/dL)	Total chol (130-200 mg/dL)	TG (0 - 150 mg/dL)	Glucose (70 - 100 mg/dl)	HbA1c (4-6.0 %)	bilirubine (0.2 - 1.2 mg/dL)	potassium (3.5 - 5.1 mg/dl)	TSH 3rd Generation (0.55	Total protein (6.6 - 8.3 g/dl)	Urea (11 - 43 mg/dl)	Creatinine (0.57-1.1 mg/dL)	eGFR (>90)	Hypertension	Ischemic heart disease (IHD)	Atrial fibrillation (AF)	Myocardial infarction (MI)	Heart failure	Stroke	Generalized atherosclerosis	Neuropathy	Retinopathy	Hypothyroidism
63	69	F		19.8		4.58	116	44	183	115	128	6.6	0.8	3.9	0.404	5.2	15	0.76	76	YES							YES		YES
64	75	F		5.2		2.64	72	33	134	145	96		0.7	4.6	0.965		34	0.82	72	YES	YES		YES			YES			
65	62	F				2.09	123	46	198	147	205	6.8		4.1	1.215		35	0.75	83	YES							YES		YES
66	69	F		3.9		2.4	80	60	159	94	106	6.1		4	1.24		34	0.8	75	YES									YES
67	72	F	0.08	10.8		53.35	86	42	165	186	144	7.5	0.2	4.9	1.12		67	1.24	45										
68	70	F	0.03			3.25	117	42	189	151	137	6.9		4.1	1.74		27	0.65	96	YES									
69	75	F	0.01			1.9	110	67	196	97	122	6.5		4.3	0.58		23	0.76	79	YES						YES	YES		
70	78	M				1.86	34	32	105	194	275	12.9		4.9	1.75		57	1.56	46	YES									
71	81	F				0.95	62	49	142	154	252	9.8		4.2	0.58		43	0.95	60	YES			YES						
72	72	F				5.4	51	39	116	129		8.4		4.1	0.53		27	0.7	87	YES		YES					YES		
73	59	F	0.01			14.35	78	43	140	94	116	5.9		4.1	0.17		22	0.79	79	YES									YES
74	68	F				2.08	195	57	275	113	141	12.9		4.6	4.783		22	0.69	90										YES
75	39	M	0.03	56.6		1.23	166	30	234	189	196	7.6		3.5	1.539		28	0.65	145										
76	59	M				<0.4	31	38	84	76	194	6.9		4.7	0.894		31	0.82	102	YES	YES		YES				YES	YES	
77	71	F				0.64	52	47	117	88	90	5.4		4.2	1.09		35	0.62	101	YES							YES		
78	68	F				7.47	101	45	166	100	97	5.6		3.7	1.38		34	0.9	66	YES	YES		YES						
79	58	M				2.15	116	21	166	146	247	6.9		4.5	0.402		29	0.55	163	YES							YES		
80	69	M				0.72	74	47	151	152	174	10.3		3.9	2.156		33	0.77	106	YES			YES	YES					
81	66	F				0.65	135	52	206	97	106	5.8		4.3	2.326		37	0.74	83	YES									
82	48	F				2.36	60	38	159	306	144	8.8		4.1	0.944		45	0.72	92	YES							YES		

83	66	F			1.24	52	50	131	147	132	8.3		4.4	0.945		57	1.06	55	YES	YES	YES				YES	YES		
84	61	F			1.78	56	42	118	99	153	7.9		3.5	1.188		32	0.65	98	YES						YES	YES		YES
85	76	M			0.82	108	54	187	127	189	8.9		4.3	0.771		42	0.96	81	YES	YES				YES	YES			
86	68	M			1.52	84	31	173	290	239	9.1		3.8	2.523		79	1.25	61	YES							YES		
87																												
88	57	F			<0.4	90	65	176	104	98	5.5		4.1	2.084		26	0.8	79										
89	75	F			1.48	146	56	216	71	100	7.9		4.5	1.229		26	0.8	74	YES								YES	YES
90	44	F			2.14	86	43	161	160	104	5.9		3.8	0.958		35	0.83	79										
91	52	F			5.3	55	47	127	123	110	6.5		4.3	0.075		40	0.67	98	YES									YES
92	74	M			19.58	39	30	82	64	240	9.8		3.9	1.747		30			YES				YES		YES	YES		
93	70	M			0.55	60	44	131	136	139	7.2		4	3.574		42	0.92	86	YES	YES					YES			YES
94	71	F			0.68					154			4.7	1.717	8	48	0.76	80	YES						YES	YES		
95	53	F			11.38	136	50	220	169	126	6.6		4.2	1.944		27	0.75	86	YES									
96	51	F			6.85	91	33	161	183	226	14.8		4.5	0.944		15	0.62	108	YES									
98	70	F	0.05	13.1	1.2	38	37	92	83	167	8.9	0.5	4.7	0.753	6.2	43	1.01	57	YES	YES				YES				
99	62	F	0.02	1290.7	5.33	65	33	118	98	193	11.5	0.7	4.7	1.146		15	0.57	114	YES							YES		
100	73	F	0.02		4.52	48	44	125	165	219	8.4	0.3	4.1	1.114	7.6	26	0.73	83	YES									
101	76	M			5.92	59	37	109	67	126	6	0.8	4.3	1.98		38	0.99	78	YES	YES	YES		YES					
102	67	F			1.04	114	45	231	359	214	8.7		4.6	0.614		55	1.1	52	YES									
103	65	F		4.5	1.64	177	54	264	167	101	7.1	0.6	4.2	1.289	6.8	37	0.63	100	YES							YES		
104	60	F	0.85	<1.9	1.16	44	52	137	206	147	7.5	0.5	5	1.33	7.3	30	0.79	79	YES	YES	YES	YES			YES	YES		YES
105	61	M			0.61	132	63	220	127	164	6.8	0.7	4.7	1.619		27	0.65	132	YES					YES	YES			
106	64	M		4.4	2.33	69	39	132	119	296	12.2		4.3	1.205		47	0.86	95	YES						YES			
107	68	M	0.09	16.6	2.36	87	37	176	262	113	5.9	1.2	4.1	1.097		39	1.2	64	YES									

Table S3. Data of patients with NfT2DM from whom serum and plasma were collected before COVID-19.

No.	Age [years]	Sex	Procalcitonin (0-0.05 ng/mL)	Troponin (0 - 15.6)	PTH (12 - 88 pg/ml)	CRP ultrasensitive (0 - 5 mg/l)	LDL (0-135 mg/dL)	HDL (>40 mg/dL)	Total chol (130-200 mg/dL)	TG (0 - 150 mg/dL)	Glucose (70 - 100 mg/dl)	HbA1c (4-6.0 %)	Bilirubin (0.2 - 1.2 mg/dL)	potassium (3.5 - 5.1 mg/dl)	TSH 3rd Generation (0.55 - 4.78)	Total protein (6.6 - 8.3 g/dl)	Urea (11 - 43 mg/dl)	Creatinine (0.57-1.1 mg/dL)	eGFR (>90)	Hypertension	Ischemic heart disease (IHD)	Atrial fibrillation (AF)	Myocardial infarction (MI)	Heart failure	Stroke	Generalized atherosclerosis	Micro-angiopathy	Macro-angiopathy	Neuropathy	Retinopathy	Hypothyroidism	Secondary hyper-parathyroidism
1	61	F	1.25		144.9	3.1	88	59	224	385	220	7.4	0.2	4.18	1.55	6.8	86	4.71	15	YES	YES	YES	YES		YES		YES	YES	YES		YES	YES
2	73	F	0.42	286.3	465.4	3.3	74	48	143	104	177	8.1	0.5	5.49	1.339	7.2	146	5.47	8	YES				YES	YES		YES	YES		YES		YES
3	92	F	0.23		81.9	12.54	118	32	190	202	78	5.6	0.4	5.59	1.363	5.5	78	5.08	8	NO	YES	YES	YES				YES	YES				
4	83	M	21.81	26.1	587.0	4.7	94	28	165	213	206	6.3	1	4.24	2.518	5.9	81	8.54	6	YES	YES	YES	YES	YES		YES					YES	
5	82	F	0.09	22.6	887.8	5.76	77	60	151	72	182	6.9	0.4	5.79	4.626	7.1	143	5.83	7	YES	YES	YES	YES	YES	YES	YES					YES	
6	87	F	0.47	29.1	606.6	43.08	150	41	239	239	92	5.8	0.4	3.93	1.004	5.7	141	3.77	12					YES								YES
7	63	M	0.11	14.7	949.1	2.92	70	34	135	138	174	7.2	0.5	6.96	11.77	7.1	166	6.59	9	YES	YES	YES		YES		YES						YES
8	80	M	0.17	30.0	777.9	27.42	45	23	79	104	174	7.3	0.7	4.66	1.303	6.9	141	5.66	10	YES	YES	YES			YES	YES						
9	66	F	0.17	156.7	1068	24.82	157	43	236	181	221	8.1	0.5	4.34	0.958	7.2	79	6.44	7	YES	YES		YES		YES	YES	YES	YES		YES		
10	49	M	0.53		2000	12.175	97	30	166	194	98	5.1	0.7	5.93		6.6	161	10.18	6	YES	YES											YES
11	74	M	0.11	15.7	951.95	6.3	79.2	57	146.8	53.4	188.5	6.4	0.6	4.65	1.14	7.5	136	2.24	31	YES	YES	YES	YES	YES		YES						
12	78	M	4.99	11.9	844.85	84.86	123	34	206	204	259	5	0.6	5.58		5.2	84	4.18	15	YES												
13	56	M	0.22	65.2	761.3	6.31	104	37	165	121	274	8	0.8	5.26	1.061	7.4	123	7.09	10	YES		YES				YES						
14	69	M	0.27	515.9	940.1	6.01	37	26	91	82	71	5.6	1.1	4.49	1.921	6.2	70	4.26	16	YES	YES		YES		YES	YES					YES	
15	83	F	0.52	350.2	1244	77.61	59	31	196	168	280	5.4	0.5	4.08	1.484	5.8	62	5.26	9	YES	YES		YES			YES						YES
16	70	M	6.31	10.8	281.5	1.25	78.7	48	152.5	152	202	6	0.6	4.35	1.207	6	92	5.28	11	YES	YES	YES				YES						
17	83	M	0.445	69.6	574.2	47.67	89	14	126	114	125	7.1	1	4.86	6.06	5.9	150	6.69	8	YES	YES	YES	YES	YES	YES	YES					YES	
18	67	M	0.21	34.8	1594.2	2.94	46	25	88	87	225	7.5	0.9	6.52		7.1	179	8.09	8	YES		YES	YES			YES				YES		
19	68	F	0.98	3622	1208.45	33.98	94	26	186	329	190	8.1	0.9	4.92	2.130	6.8	163	6.185	7	YES	YES		YES		YES	YES						
20	76	M	0.2		2000	4.22	63	48	148	51	103	6.1	1.8	4.9		7.4	147	7.24	8	YES		YES		YES		YES						
21	67	M	0.41	44.3	252.65	13.04	93	35	153	164	151.5	5.8	0.5	5.18	1.357	6.9	145	8.31	7	YES												
22	67	F		16.9	442.4	15.57	74	35	141	162	131	6.9	0.5	4.63	1.139	6.55	87	4.38	13	YES		YES										
23	73	F	0.28	37.9	1873.2	18.11	64	43	141	173.7	110	6.5	0.4	5		7.3	169	8.95	5	YES		YES			YES							YES
24	52	M	2.45	55.1	888.9	7.91	94	24	154	180	194	10.8	0.8	5.9	2.321	6.9	161	8.57	7	YES	YES	YES	YES			YES			YES	YES		YES

25	82	F	0.17	21.2	744.7	29.14			149	135	150	5.9	0.9	4.53	3.342	4.5	104	3.69	12	YES		YES		YES	YES							
26	70	F	0.22	80.6	233.5	26	59	45	163	132	193	8.7	0.8	3.28	2.893	5	48	2.05	25	YES	YES		YES	YES		YES						YES
27	68	M	0.34	3347.6	438.4	31.06	48		106	114	163	6.9	0.4	5.7	0.907	6.5	191	5.77	10	YES			YES									
28	68	M	0.373		364.1	2.33	102	34	156	102	137	5.9	0.4	4.61	4.287	7.1	78	5.7	12	YES	YES		YES							YES		YES
29	70	F	0.48	17.9	404.2	4.61	75	65	156	69	188	10.9	0.5	4.83	1.133	5.5	74	2.185	20.5			YES		YES						YES		
30	64	M	0.26	87.0		85.44	108	62	223	266	133	6.8	1.4	4.09	0.063	5.1	31	2.44	29	YES		YES		YES		YES						
31	68	F		15.3	334.8	0.78	233	101	377	214	152	6.5	0.2	5.33	1.785	4	104	4.905	9	YES												YES
32	41	F	0.94	31.4	1952.6	50	149	45	221	238	358	8.4	0.5	5.07	0.451	6.5	109	5.84	8	YES				YES		YES				YES		YES
33	63	M	0.12	3	304.1	31.12	57		139	141	113	8	0.3	4.6	1.153	6.5	91	3.91	17	YES												
34	73	F	0.62	112.7	2077.2	58.55	83	42	151	131	127	6	0.5	4.3	2.241	4.3	142	4.19	11	YES	YES	YES				YES						YES
35	64	M	0.26		302.4	73.19	47	34	96	74	114	7.5		5.21	3.248	6.4	105	2.38	29	YES	YES		YES	YES	YES	YES	YES	YES		YES		
36	54	M	0.99	16.6		5.92	120	31	201	249	182	5.6	0.8	4.37	3.716	6.3	151	2.22	33	YES				YES								
37	51	M	1.82	35.7	254	142.92	48	35	108	123		9.2	0.6	6	3.161	5.3	113	2.50	30	YES	YES	YES		YES		YES						
38	73	F	0.28	111.3	278.3	72.85	160	49	237	139	107.7		0.8	4.71	1.274	5.8	110	3.31	14.4													
39	66	F			553	3.44		40	175	468	207	7	0.6	4.74	1.024	6.5	148	5.63	8		YES										YES	
40	63	M	0.06	59.1	312.8	4.945	77	30	132	126	105		1.2	4.43	0.73	6	186	3.08	22		YES		YES	YES								
41	89	F	8.56		205.3	42.25	66	53	133	71	112	6.2	1.1	4.7	2.384	7.9	88	2.36	21		YES	YES	YES		YES							
42	77	F	0.2	11		4.24	76	48	158	168	214	10.7	0.5	4.7	0.756	6.8	90	4.25	11	YES												
43	88	F	0.22	24.4	208.2	36.14	49	43	121	147	128	5.7	0.5	5.17	3.087	5.2	111	2.84	17	YES		YES		YES								
44	77	F	0.3	18.6	282.1	7.8	70	52	137	75				6.09	1.86	7.3	101.5	6.65	6													
45	57	M	0.14		938.2	2.45	91	49	180	200	122	7.1	0.5	6.37	0.836	7.3	155	9.55	6	YES	YES	YES		YES								
46	81	F	0.04		261.3	6.45	185	44	273	221		6.9	0.6	5.23	2.383	7.2	97	1.99	26	YES	YES		YES		YES	YES					YES	YES
47	63	M				2.07	48	34	101	94	106	6.6	0.5	4.63		5.8	98	3.32	20	YES	YES		YES	YES	YES							
48	33	F			230.1	0.49	145	74	276	283	87		0.3	5.32	1.802	6	42	2.01	30	YES										YES		YES
49	29	F	0.05			1.72	87	59	180	171	189	8	0.5	3.76	1.001	6.3	167	1.52	43	YES												YES
50	71	M		38.2	105.2	14.85					167	6.7	0.6	4.83	2.259	7.5	152	5.27	12			YES		YES	YES		YES	YES	YES	YES		
51	69	M	0.16			1.3	74	47	143	108			0.4	4		4.9	73	4.88	13	YES						YES						

Table S4. Data of patients with NfT2DM from whom serum and plasma were collected after COVID-19.

6	AGE [years]	sex	Procalcitonin (0-0.05 ng/mL)	Troponin (0 - 15.6)	PTH (12 - 88 pg/ml)	CRP ultrasensitive (0 - 5 mg/l)	LDL (0-135 mg/dL)	HDL (>40 mg/dL)	Total chol (130-200 mg/dL)	TG (0 - 150 mg/dL)	Glucose (70 - 100 mg/dl)	HbA1c (4-6.0 %)	bilirubine (0.2 - 1.2 mg/dL)	potassium (3.5 - 5.1 mg/dl)	TSH 3rd Generation (0.55 - 4.78)	Total protein (6.6 - 8.3 g/dl)	Urea (11 - 43 mg/dl)	Creatinine (0.57-1.1 mg/dL)	eGFR (>90)	Hypertension	Ischemic heart disease (IHD)	Atrial fibrillation (AF)	Myocardial infarction (MI)	Heart failure	Stroke	Generalized atherosclerosis	Bilateral diabetic foot	Chronic limb ischemia	Neuropathy	Retinopathy	Hypothyroidism
1	85	F	0.3	48.7	1151.5	17.47	150	47	236	197	104		0.4	5.3	1.179	6.2	90	3.33	14	YES	YES			F	YES	YES	tak				
2	66	M	1.03	49	144.2	233.		23			328	9.1	0.3	5.8			143	7.53	8	YES	YES				YES			YES			YES
3	74	F	69.51	84.9		223.4					78		4.5	5.9		4.500	104.000	3.78	13	YES											YES
4																															
5	40	F	0.5			22.43	89	46	153	91	88		0.4	4	1.715	7.4	55	2.42	24	NO											
6	81	F	17.2	70.1	239.2	52.17					152			4		5.3	97	3.8		YES	YES				YES						YES
7																															
8	71	M	0.22	530,15	489.4	30,9	64	61	141	78	106	4,9	6.5	4.4	1.602	6.5	36	4.38	14	YES											
9	42	M	0.24		1253.5	18.65					67		0.6	5.90		8.1	110	6.13	11	YES											YES
10	55	F	0.13	200,5	934.6	4,2	40	44	102	91	85 (07.2021r.)		0,7	6.1	0.736	5.6	121	3.72	13	YES	YES			YES				YES			
11																															
12	51	F	0.69			1.6	230	51	334		87	5.8	0.3	3.81	1.5	3.8	50	3.23	16												YES
13	56	M	0.13	18.2	226.3	7.64	70	28	128	150	190	5.4	0.7	7.4	2.286	6.3	125	7.4	8	YES	YES										
14																															
15	62	M	0.74	164.5	2034	7.51	68	32	131	153	180	5.8	0.8	5.6	1.15	6.5	175	5.1	12	YES	YES			YES			tak	YES			
16	34	M	0.23	<1,9	1178.	9.12	116.0	36	171	97	89		0.8	4	0.799	7.2	101	10.05	6	YES											
17	69	F	0.23	168.9	411.1	15.21	136	64	219	93	94	5.4	0.6	3.80	2.508	6.9	90.25	4.33	11	YES		YES									YES
18																															
19	36	M	3.25	92.3	507.5	67.95	68	19	108	104	93		1.3	5.7	0.651	6.4	91	5.96	12	YES											YES
20	54	M	0.08	47.3	882.8	3.61	151	41	205	66	87	5.4	0.4	5.5	1.189	6.2	235	10.08	6	YES											
21	61	M	0.94	10.9	239.7	6.98	65	116	195	70	89	4.9	0.3	5.3	0.522	4.8	45.5	5.42	11	YES	YES			YES							
22																															
23																															
24	88	F	0.11	12.7	359.1	43.86	58	31	111	108	71		0.6	5.1	3.125	7.8	66.5	4.88	9	YES	YES	YES			YES					YES	
25	88	M	0.68		467.9	170	94	24	150	160	102		0.8	3.4	2.101	6.7	60	3.61	17	YES						YES					
26	77	M	0.26	18.6	211.4	4.33	123	36	198	193.0	137		0.9	5.1		5.2	56.5	5.15	12	YES											
27	66	M	0.81	4	100.1	116.65	84	33	181		112	5.6	0.3	4.7	1.841	5.9	116	10.87	5	YES	YES	YES									YES
28																															
29	21	F	0.8	73.3	1392.8	<0,4	148	48	233	< 3	108		0.5	5.3	0.191	4.7	97	7.92	7	YES											YES
30																															YES
31	89	M	0.05	21.2	400.1	30.99	101	39	149	46	76		0.6	4.5	0.824	5.9	77	3.24	19	YES	YES	YES		YES		YES					YES
32	65	M	0.74	26.5	543.9	1.44	71	62	178	82	141	5.2	0.3	5.5	1.366	6.3	20	3.81	17	YES							tak		YES		
33																															
34	80	F	0.230	7.3	563.8	0.72	131	55	212	122	89	5.1		4.8	3.844	6.6	64	4.3	11	YES											
35	83	F	0.15	17.9	1249.3	3.22	110	49	173	68	76			5.5		7.5	46	8.1	5	YES		YES							YES	YES	YES
36	82	F	0.21	15.0	169.4	8.65	82	67	171	110	243	7.5	0.5	5.1		5.7	44	3.85	12	YES							tak				YES
37																															
38	29	M	0.52		154.4	14.15	42	33	80	25	78		0.6	4.4	1.669	7.3	73	5.19	14	YES											YES
39	84	F	0.40	313.4	1514.2			44	113	101	153	7.6	0.3	5.1	2.753	7.1	35	6.41	7	YES											
40	42	M	4.46	46.9	1905.4	128.48	46	45	107	82	65		0.4	5.4	2.55	6	163	8.64	7	YES											YES
41	54	F	0.1		1962.2	23.18	105	26	167	181	85		0.5	4.7	1.249	6.2	20	5.35	9	YES											

**42**	60	M	0.34	23.3	365	2.18	90	25	125	51	176	7.9	0.8	4.9	0.653	5.3	59	5.8	11					YES	YES			
**43**	32	M	0.2	5.8	756.4	<0,4	37	43	101	105	91		0.3	4.5	1.886	6.7	99	9.21	7	YES								
**44**	35	M		19.2	654.1	41.66	71	37	122	70	108	5.8	0.6	4.8	2.159	5.2	133	10.43	6	YES								YES
**45**	73	M	0.23	33.4	417.3	2.31	154	47	188	104	80.0	5.6	0.4	4.3		6.5	131	5.29	11	YES					YES			
**46**	75	M	0.09	21.8	36.8	16.75	76	51	143	82	110		0.5	3.2	1.291	6.9	77	3.56	18	YES		YES			YES			
**47**	48	M	0.66	7.9	1401.5	18.77	81	35	133	86	63		1.1	6.8	0.364	7.6	107	10.42	6	YES	YES	YES				YES		
**48**	56	M	0.26	72.9	261.1	78.24	53	15	90	111	203.5	8.7	1.3	5.2	4.449	7.3	64	4.08	16	YES	YES	YES		YES				
**49**	48	M	0.52	4.7	881.4	2.13	58	50	149	207	104		0,4	5.1	2.253	6.4	89	7.12	9	YES								
**50**	70	M	0.68	8.8	628.2	6.18	61	49	132	111		5.1	0.8	4.4	2.678	7.1	32.5	2.43	28	YES								
**51**	54	F			2035.4											6.8	217	10.27	4	YES	YES							
**52**	65	M		14	953.2	5.51	146	38	234	251	76	5,4	0.7	5.3	0.911	7.1	72.5	10.66	5	YES								
**53**	47	M	0.12	3.8	592.8	1.64	70	36	140	172	123	6.1	0.3	6.7	0.4204	5.8	117	4.7	14	YES								
**54**	65	F	2.45	40.5	112.2	56.19	33	31	90	132	62		0.4	4	2.3677	3.2	97	6.17	7	YES					YES			
**55**	41	M	0.53	4	462.5	67.38	91	40	141	51	93			4.4		6.4	116	5.78	12	YES								

Table S5. Values of selected basic diagnostic and anthropometric parameters of the control group.

No	Age [years]	SEX	RBC [mln/µl] K:3,8-5,8 M: 4,5-6,5	HGB [g/dl] K:11,5-16 M: 13-17	HCT [%] K:37-47 M: 40-54	MCV [fl] 80-100	MCH [p/g] 27-32	MCHC [g/dl] 32-36	PLT [tys./µl] 150-500	WBC [tys./µl] 4-10	NEU [%] 50-80	LYM [%] 25-50	MON [%] 2-10	EOS [%] 0-5	BAS [%] 0-2	HbA_{1C} [%] <6.0	Creatinine [mg/dl] 0-2	Total Cholesterol [mg/dL] 80-190	HDL mg/dl	LDL mg/dl (<145)	Triglyceride [mg/dL] 0-150	Total protein [g/dL] 6 – 8
1.	50	M	4.95	14.9	43.8	88	30.1	34	278	5.9	67.3	24.7	5.7	2	0.3	5.1	1.13	199	82.5	95.3	104	6.8
2.	48	F	5	13.6	42.2	84	27.2	32.3	251	5.5	48.9	38.7	9	3	0.4	5.6	1.14	222	54.6	132.7	172	7.4
3.	46	F	4.54	14.1	41.4	91	31.1	34.1	202	6.6	60.5	30.4	6.7	2.1	0.3	5.3	1.06	199	74.4	93.46	156	7.5
4.	71	F	4.41	14.1	41.9	95	31.9	33.5	220	6.1	65.8	25.1	5.2	3.5	0.4	5.9	1.08	209	81.2	104.5	116	7.5
5.	57	M	4.77	15.1	45.1	94	31.8	33.6	169	5.1	47.5	43.2	5.3	3.6	0.4	5.6	1.14	160	61.3	89	47	6.4
6.	46	M	5.26	16.7	48.4	92	31.7	34.4	159	5.8	59.3	29.6	7.8	2.6	0.7	5.6	1.15	192	26.9		768	7.4
7.	51	M	4.96	15.3	44.7	90	30.8	34.2	164	5.9	70.3	20.9	5.6	3	0.2	5.2	1.1	184	50.3	109.42	123	7.2
8.	46	M	5.37	15.7	47.2	88	29.3	33.3	239	6.8	57.9	29.9	6.1	5.6	0.5	5.9	0.95	166	50	89.8	132	6.6
9.	52	F	5.25	15.4	46.4	88	29.3	33.1	202	7.4	58.1	34	5.3	2.2	0.4	5.6	1	272	81.4	170.5	100	7.7
10.	54	M	5.26	15.7	46.6	89	29.8	33.6	283	7.1	54.3	38.1	4.9	2.4	0.3	5.4	1.21	238	59	141.46	186	7.5
11.	53	M	4.95	16.2	47.7	95	32.7	34	308	7.2	60.8	29.4	5.5	3.9	0.4	5.9	0.82	170	51.6	101.58	82	7.1
12.	63	F	4.42	13.5	39.7	90	30.5	34	210	5.4	61	30	5.8	3	0.2	5.2	1.09	213	42.7	122.08	239	7
13.	48	M	4.76	15	43.6	92	31.5	34.4	248	6	51.7	37.8	7	3	0.5	5.5	0.98	221	39.2	136.44	225	6.6
14.	46	F	4.51	14.6	43.1	96	32.4	33.8	248	10.1	64.4	27.4	6.1	1.6	0.5	5.6	1.02	221	63.1	114.06	219	7.5
15.	46	M	5.14	15.8	45.8	89	30.6	34.4	192	6.5	54.6	33	8.7	3.2	0.5	6.1	1.06	287	47.2	194.94	222	7.7
16.	50	M	5	15.2	45.2	90	30.4	33.7	222	5.5	66	25.2	5.2	3.3	0.3	5.6	1.1	157	39	94.22	117	7.4
17.	51	F	4.05	13	38.5	95	32	33.7	263	8.2	70.1	22.7	4.3	2.6	0.3	5.3	1.03	211	79.1	110.22	110	7.7
18.	46	F	4.45	14.4	41.7	94	32.4	34.7	234	5.5	55.8	36.3	6	1.6	0.3	5.3	1.2	171	61	89.94	99	7.6
19.	61	M	4.87	15.9	46.1	95	32.7	34.6	172	7.1	53.4	32.1	10.7	3.3	0.5	5.7	1.29	143	37.3	82.38	118	7
20.	58	F	4.71	14.2	41.6	88	30.1	34.1	265	6.7	62.9	22.7	8.7	5.2	0.5	5.8	0.59	243	50	131.66	305	7.4
21.	65	M	4.48	13.8	41	92	30.9	33.7	308	7.3	61.7	24.3	11.1	2.5	0.7	5.6	1.2	238	66.6	144.12	138	7
22.	64	F	4.36	13.8	40.5	93	31.7	34.1	252	5.2	42.7	47.8	5.4	3.8	0.3	5.8	1.03	239	68.3	131.18	197	7
23.	54	M	4.72	15.7	45.3	96	33.3	34.7	202	5.9	60.5	28.5	6.7	3.9	0.4	5.4	1.15	251	63.2	138.8	246	6.8
24.	65	M	4.68	15.3	44	94	32.7	34.9	159	6.9	52.6	33.5	12	1.4	0.5	5.6	0.94	272	24.5	24.02	1350	7.4
25.	48	M	4.8	14.8	43.1	90	30.9	34.4	182	5.7	44.4	44.3	6.3	4.7	0.3	5.1	1.33	200	45.2	121.1	170	7.2
26.	53	M	5.19	16.7	47.9	92	32.2	35	215	5.3	50.9	38.8	5.1	4.8	0.4	5	1.1	188	43.5	92.26	263	7
27.	47	M	5.44	15.4	45.4	84	28.3	33.9	219	8	53.5	35.9	5.5	4.7	0.4	5.2	1.48	169	55.5	93.68	99	7.2

28.	47	M	5.13	13.7	42.3	83	26.7	32.3	309	6.7	47.3	42.1	7.4	2.8	0.4	6	0.99	208	45.5	127.78	172	7.2
29.	55	F	4.55	13.9	40.6	89	30.5	34.1	358	7.6	55.4	35.1	5.9	3.2	0.4	5.4	0.78	215	56.6	114.26	220	7.3
30.	50	F	4.84	14.9	43.3	90	30.7	34.3	266	7.2	61.8	30.9	4.6	2.3	0.4	5.3	1.07	259	61	159.18	192	7.7
31.	51	F	5.08	14.8	43.9	86	29.1	33.7	337	6.9	72.8	20.7	4.6	1.5	0.4	5.3	0.99	210	41.1	108.12	304	7.1
32.	54	M	5.18	14.3	43.4	84	27.6	32.9	241	7	67.4	24.6	5.6	2	0.4	5.8	1.22	209	41.2	128.6	197	7.9
33.	46	M	5.06	16.5	48.3	95	32.6	34.1	242	5.2	57.1	34.7	5.6	2.4	0.2	5.3	1.01	217	52.7	133.2	154	7.4
34.	65	M	5.05	15.3	45.1	89	30.3	33.9	290	8.2	53.6	35.6	7.5	2.5	0.6	5.9	0.94	225	37.2	125.78	311	6.7
35.	62	M	4.71	14.6	42.4	90	31	34.4	219	8.9	63.2	29.6	5.2	1.7	0.3	5.6	1.06	203	40.9	126.02	182	7.1
36.	51	F	4.74	14.4	42.5	90	30.4	33.9	304	6.6	58.7	31.2	6.4	3.4	0.3	5.6	1.06	275	56.9		409	8.3
37.	47	F	3.99	13.3	38.9	98	33.2	34	251	5.1	53.1	40	4.6	1.7	0.6	5.4	0.69	225	46.9	149.8	142	7.4
38.	48	M	4.82	14.6	43.6	90	30.3	33.5	347	8.6	54.1	30.4	8.3	6.5	0.7	5.5	1.03	252	41.1	156.38	271	7.3
39.	50	M	4.66	14.9	44.6	96	31.9	33.4	294	8.4	43.1	39.8	12.3	4.2	0.6	5.4	0.96	267	58.9	172.26	177	8.4
40.	58	M	5.56	15.6	46.5	84	28.1	33.6	268	5.5	59.2	30.9	6.9	2.8	0.2	5.4	0.88	200	43	127.1	150	7
41.	47	F	4.57	12.5	39.2	86	27.3	31.9	278	5.2	55.8	33.3	7.2	3.4	0.3	5	0.88	171	68.7	90.58	59	7.3
42.	49	F	4.5	14	42.1	94	31.2	33.3	243	7.4	57.2	28.2	7.3	6.8	0.5	5.3	0.62	226	63.9	134.58	137	7.7
43.	54	M	4.83	15.4	44.6	92	31.9	34.6	165	6.3	59.8	31.6	4.8	3.4	0.4	5.3	1	209	32.9		480	7.4
44.	57	M	5.07	15.5	45.3	89	30.5	34.1	326	6.2	65.3	24.1	8.2	2	0.4	5.3	0.88	206	54.4	151.7	129.5	111
45.	47	M	4.45	14.3	42	94	32.1	34	232	6.4	63.9	27.5	6	2.3	0.3	5.2	0.95	192	70.2	91.64	151	7.7
46.	53	M	5.59	15.6	47.7	85	27.9	32.7	306	6	58.3	27.8	11.2	2.3	0.4	6.2	0.91	274	54.3	187.52	163	7.9
47.	47	M	5.4	14.9	45.2	84	27.6	33	213	7.6	52.1	36.5	7.8	3.1	0.5	5.4	1.03	234	41.5	133.6	296	7.5
48.	55	M	4.5	14.9	43.5	97	33.1	34.3	360	4.9	51.9	33.1	9	5.7	0.3	5.6	1.17	254	43.1	161.14	247	7.5
49.	60	M	4.5	14.3	42.1	94	31.8	34.1	213	7.5	52.6	39.4	5.2	2.4	0.4	5.5	0.99	262	56.3	172.34	168	7.5
50.	50	M	5.13	15.9	47.5	93	31.1	33.6	275	6.5	52.3	39.2	6.2	2.1	0.2	5.4	1.09	215	38.4	131.66	226	7.4
51.	49	F	4.28	12.9	38	89	30.1	33.9	336	7.1	56.2	34.6	7.2	1.7	0.3	5.5	0.98	242	51.3	190.5	147.94	213
52.	52	F	4.13	13.3	39.4	95	32.3	33.8	199	5.4	46.5	44.1	5.8	3.4	0.2	5.3	0.82	160	79.1	51.58	148	7.1
53.	52	M	4.99	14.5	43.4	87	29	33.4	213	4.4	50.5	41.5	4.3	3.3	0.3	5.4	0.92	172	39.2	111.78	107	7.2
54.	47	M	5.1	16.9	49.6	97	33.1	34	228	5.9	49.5	40.7	6.5	2.9	0.4	5.1	0.96	242	54.9	152.5	175	7.4
55.	48	M	4.8	14.8	43.6	91	30.8	33.9	217	5.3	51.8	33.4	8.8	5.7	0.3	5.5	0.92	230	63	140.44	132	8
56.	50	M	4.83	15.3	45.8	95	31.6	33.4	168	5.8	59.9	28.6	7.6	3.6	0.3	5.7	0.94	230	51.5	114.34	323	7.7
57.	46	F	4.17	12.4	38.3	92	29.7	32.4	367	7.6	58.8	31.7	6.7	2.4	0.4	5.2	0.77	244	95.3	112.82	180	8.2
58.	45	M	4.68	15.1	45.3	97	32.2	33.2	161	3.8	53.6	36.2	7.5	2.4	0.3	5.3	0.95	206	55.6	155.5	173	7.4

59.	49	M	4.66	15.5	45.7	98	33.2	33.8	215	4.9	49.5	39.3	8.8	2	0.4	5.2	0.86	158	35.4	63.28	296	6.6
60.	52	M	4.99	17	49.7	100	34.1	34.2	254	4.4	53	34.6	8.5	3.4	0.5	5.1	0.84	207	53.4	127.68	129	7.4
61.	49	M	5.16	13.5	41	79	26.1	32.8	304	7	55.7	34.8	6.4	2.8	0.3	5.6	0.96	243	37.5	164.28	204	7
62.	52	M	4.65	15.2	44.9	97	32.7	33.9	223	3.9	47.9	43	5.1	3.6	0.4	5.2	0.91	228	61.3	144.88	108	6.9
63.	51	F	4.34	13.4	39.2	90	30.8	34.1	276	5.1	50.7	40.4	5.7	3	0.2	5.3	0.74	183	71.2	94.42	88	6.8
64.	47	M	5.01	15	44.2	88	29.9	33.8	251	5.1	57.5	35	4.1	3.1	0.3	5.5	1.06	186	51.5	96.78	191	7.1
65.	45	M	4.58	14.5	42.3	92	31.5	34.2	353	5.5	54	36.7	6.9	1.9	0.5	5.3	0.96	161	39.5	90.68	155	7.7
66.	50	F	4.81	14.7	44.3	92	30.6	33.2	185	6.6	61.7	25.9	4.4	7.7	0.3	5.6	0.72	259	52.9		461	7.5
67.	49	M	4.99	16.2	47.6	96	32.5	34	200	6	55.1	35.9	5.7	3	0.3	5.7	0.94	266	43.9	164.92	284	7.2
68.	62	M	4.86	15.6	46	95	32.1	33.9	241	6.7	56.3	34.2	7.8	1.4	0.3	5.6	0.88	244	54.9	138.82	253	7.2
69.	48	M	5.08	15.6	45.6	90	30.7	34.2	223	4.9	64.2	29.3	4.2	2	0.3	5.4	0.84	195	31.5	119.62	220	6.8
70.	50	F	4.71	13.2	39	83	28.1	33.9	278	4.8	59.9	31.7	5.5	2.6	0.3	5.3	0.7	191	74.9	84.38	158	8.1
71	47	M	4.81	14.8	44.4	92	30.7	33.3	258	9.7	62.3	28.7	4.7	4	0.3	5.3	0.75	206	63.6	116.82	129	6.9
72.	46	M	4.83	15.1	44.8	93	31.3	33.7	204	4.2	62.3	27.8	6.1	3.5	0.3	5.3	1.02	227	49.5	141.26	183	7.6
73.	55	M	4.55	15.2	43.9	96	33.4	34.6	245	5.5	67.3	25.7	4.7	2.1	0.2	5.2	1.28	219	62.5	120.94	177	8.1
74.	48	M	5	14.8	44.2	88	29.6	33.5	204	6.1	48.5	41.8	4.8	4.5	0.4	5.7	1.28	162	41.4	100.14	100	7.7
75.	45	M	5.43	15	45.5	84	27.6	33	253	5.2	46	38.5	8.4	6.8	0.3	5.5	1.21	174	40.6	100.74	164	7.3
76.	49	F	4.72	14.2	42.3	90	30	33.4	229	6.8	59	33.4	5.5	1.8	0.3	5.1	0.96	201	65.6	108.86	134	7.6
77.	46	F	4.37	14.6	41.8	96	33.5	35	239	9.6	60	29.7	6.4	3.4	0.5	5	0.83	264	48.9	172.3	213	6.9
78.	45	F	4.85	14.7	44	91	30.3	33.4	273	8.9	48.8	43.3	5	2.4	0.5	6.1	1.05	228	52.1	152.86	114	7.8
79.	46	M	4.93	14	41.8	85	28.4	33.5	181	7.4	63.4	30.5	3.8	2	0.3	5.2	1.29	216	61.6	153.9	135.76	91
80.	50	M	4.27	14.1	42	98	33	33.6	247	6.4	54	35.1	7.2	3.4	0.3	5.1	1.37	218	49.5	123	229	8.2
81.	53	M	4.52	14.9	44.6	99	33	33.4	197	7.2	55.5	33.8	8.5	1.7	0.5	5.5	1.15	207	61.4	145.9	108.34	188
82.	52	M	5.24	16.5	48.9	93	31.4	33.7	280	8.2	66	25.1	7.1	1.1	0.7	5.4	1.36	300	58.9	211.44	149	7.5
83.	47	M	4.85	16.2	47.1	97	33.4	34.4	329	4.5	59	27.9	10.4	2.1	0.6	4.9	1.06	161	53.4	71.68	180	8.1
84.	47	F	4.48	14.4	42	94	32.2	34.3	347	5.3	44.7	45.1	7	2.8	0.4	6.1	0.77	235	43.1	125.56	333	7.9
85.	46	M	5.68	14.1	44.4	78	24.8	31.8	259	6.8	51.9	35	10.4	1.9	0.8	5.7	1.17	154	33.2	93.96	134	7.4
86.	48	M	4.97	15.9	47.5	96	32.1	33.6	253	7.5	51.1	36.4	6.6	5.4	0.5	5.6	1.23	220	62.9	122.74	170	170
87.	46	M	5.02	16	47.9	95	31.9	33.5	251	6.9	60	28.8	8.8	1.9	0.5	5.2	1.16	219	39.2	138.28	205	7.5
88.	48	M	5.65	16.3	48.9	87	28.9	33.4	214	7.9	60.6	25	9.4	4.5	0.5	5.5	1.13	167	36.7	101.8	140	7.1
89.	58	M	4.7	14.1	42.1	90	29.9	33.4	191	4.8	61	28.5	5.4	5	0.1	5.3	1.1	149	49.2	56.3	215	7.1

90.	45	F	4.64	13.9	41.3	89	29.8	33.6	279	8.2	59.4	31.7	4.2	4.5	0.2	5.1	1.23	169	38.9	96.04	170	7.5
91.	45	M	4.75	13.8	40.7	86	29.1	34	241	5.4	67.1	26.8	4.4	1.4	0.3	5.5	1.05	193	54.4	118.22	99	7.2
92.	54	M	5.23	15.5	45.7	87	29.7	34	254	8	71.1	21.7	5.4	1.4	0.4	5.5	1.28	217	45.4	122.58	243	7.1
93.	51	M	5.13	15.2	43.9	86	29.6	34.6	248	5.4	48.1	39.8	6.7	5.1	0.3	5.2	1.3	251	54.1	147.7	245	7.6
94.	47	M	4.82	14.9	45.5	94	30.8	32.6	239	8.7	53.4	34.7	7	4.4	0.5	5.7	1.64	183	33.8	77.74	359	7.7
95.	46	M	5.31	15.9	47.3	89	29.9	33.5	314	7.9	60.7	27.5	6.4	4.9	0.5	5.2	1.29	191	38.2	116.68	180	6.9
96.	53	M	5.2	14.6	44.4	85	28	32.8	238	5.3	68.4	21.6	6.5	3.3	0.2	5.6	1.2	251	51.1	166.44	169	6.9
97.	45	M	4.48	14.3	43	96	31.9	33.2	249	7.8	61.5	28	7.3	2.9	0.3	5.3	0.81	168	58.9	86.56	114	6.6
98.	52	M	4.3	13.9	41.4	96	32.4	33.6	196	9	68.1	21.3	6.1	4.1	0.4	5.2	1.15	178	59.8	105.1	63	7.1
